# Evaluating the Incorporation of *Myrtus communis* L. Leaves Infusion in Alginate-Based Films and Spheres to Enhance the Oxidative Stability of Oil-in-Water Emulsions

**DOI:** 10.3390/polym16050649

**Published:** 2024-02-28

**Authors:** Nisserine El Hammadi, María Pilar Almajano, Maria Vicenta Pastor, Idoia Codina-Torrella

**Affiliations:** 1 Chemical Engineering Department, Universitat Politècnica de Catalunya, Av. Diagonal 647, 08028 Barcelona, Spain; elhammadi.nisserine@gmail.com (N.E.H.); maria.vicenta.pastor@upc.edu (M.V.P.); 2 Agri-Food Engineering and Biotechnology Department, Universitat Politècnica de Catalunya, Esteve Terrades 8, 08860 Castelldefels, Spain

**Keywords:** *Myrtus communis* L., antiradical, antioxidant, oil-in-water emulsion, alginate film, spheres

## Abstract

*Myrtus communis* L. is a species of the Myrtaceae family that is found in the Mediterranean region, and it is traditionally recognized for its importance and different uses. The objective of this study was to determine the effect of *M. communis* L. leaf extract (MCLE), which was incorporated directly into alginate spheres and films, on preserving oil-in-water emulsions from oxidation. For this purpose, the solvent extraction (with ethanol at 40, 60, and 80%) of the antioxidant compounds was optimized (total phenolic compounds (TPCs) and total flavonoid content (TFC)) along with the scavenging activity. The best condition for the extraction corresponded with 60% ethanol (MCLE60), with a TPC of ~66.06 g GAE/L and a TFC of ~18.91 g QE/L, which was selected for use in the following assays. MCLE60 showed a considerable radical scavenging activity (24.85 mmol TE/L in FRAP, 28.75 mmol TE/L in DPPH, 30.61 mmol TE/L in ABTS, and 14.94 mmol TE/L in ORAC), which was probably due to its content in the phenolic compounds arbutin (122.08 mg/L), epicatechin (73.89 mg/L), sinapic acid (51.85 mg/L), and gallic acid (36.72 mg/L). The oil-in-water emulsions with the MCLE60 spheres showed the best oxidative stability (TBARS ~2.64 mg MDA/kg of emulsion, PV ~35.7 meq hydroperoxides/kg of emulsion) in comparison to the control. The film was also able to protect the emulsion from oxidation for more than a week at 30 °C (TBARS ~1.9 mg MDA/kg of emulsion). The alginate films with MCLE60 presented an important release of phenolic compounds in water and acetic food simulants, while in both ethanol simulants, the release of TPC remained more stable over time. Thus, this study highlights the potential uses of MCLE as a natural ingredient for emulsion oxidative preservation and the production of alginate delivery systems (spheres and films).

## 1. Introduction

Aromatic herbs have long been used as important sources of natural medicines and cosmetics. Throughout history, humans have utilized various compounds found in plants, which are highly valued for their antioxidant, antibacterial, anticancer, and anti-inflammatory properties [1]. Plants are essential to maintaining human health and improving the quality of life in general. They are widely used in food preparation, drinks, cosmetics, additives, and medicine [2]. Nowadays, the media’s focus on the health benefits of antioxidants in our diet underscores the significance of these natural sources [3]. Antioxidants are substances that are widely utilized in different formulations (food, pharmaceuticals, etc.) to prevent reactions with oxygen in the surrounding environment; they can react with free radicals to generate stable or meta-stable products, thus stopping the oxidation reaction [3]. These groups of compounds neutralize free radicals and reactive oxygen species (ROS) in the cell and lower or stop other molecules from oxidizing [4,5]. 

Free radicals generated in a redox reaction have the potential to start damaging chain reactions, which can be halted by antioxidants by oxidizing free radicals, thereby reducing their activity [6]. 

Plant enzymes such as superoxide dismutase and catalase play a direct role in inhibiting free radical generation within plant cells. Meanwhile, secondary compounds like polyphenols and carotenoids function as potent scavengers of free radicals, thereby safeguarding plant cells from oxidative damage [7,8].

Plants can be used in a variety of ways, including in the extraction of essential oils or the extraction of substances like vitamins and antioxidants, which can be extracted using several organic solvents like methanol and ethanol [9]. Phenolic compounds, which are prevalent in various plant species, exhibit significant structural diversity as secondary metabolites. They may be present in the form of glycosides or aglycones, either as free-bound compounds within a matrix or in polymerized or monomer structures [10]. They are produced in plants via the shikimic acid pathway and pentose phosphate. Ranging from simple phenolic molecules to highly polymerized compounds, they include phenolic acids, flavonoids, tannins, coumarins, lignin, and quinones, among others [11]. Recently, there has been a growing increase in the use of plant extracts, including natural elements like phenolic compounds and vitamins, in delivery systems. This evolution has been fueled by a preference for natural sources over synthetic alternatives. Plant extracts are emerging as potent natural oxidative stress defenders with a variety of bioactive components. They provide safer substitutes for synthetic antioxidants despite drawbacks including unpredictability and solubility restrictions [12,13]. However, achieving optimal effectiveness requires precise selection, extraction, and formulation. They have a lot of potential benefits in reducing oxidative stress, but further study is needed to ensure consistency and increase potency [14].

Plant extracts could be incorporated into spheres and films, among others. Films provide a natural way for food packaging and protection, and spheres present a good option to stock the natural substances extracted from plants inside the sphere’s skin and allow the diffusivity of the natural substances to the exterior environment [15,16]. This dual functionality preserves food quality, extends their shelf life, and aligns with consumer preferences for sustainable packaging. Ongoing research aims to optimize formulations for more effective plant extract-infused biodegradable films [17,18]. Likewise, investigations of these plant extracts with antioxidants, introduced in oil-in-water (O/W) emulsions and incubated at constant temperature, have evidenced their ability to retard lipid oxidation [19,20]. They act by protecting lipids from oxidation initiators or hindering the propagation phase, known as chain-breaking antioxidants [21]. *Myrtus communis* L., also known as Myrtle and belonging to the Myrtaceae family, is an evergreen bush with an average height ranging from 1 to 5 m. This plant is naturally found, particularly flourishing in the Mediterranean region [22,23]. Various parts of the plant have found applications in the food (i.e., as flavorings) and cosmetic industries, as well as in traditional folk medicine. In traditional folk medicine, a decoction made from the leaves and fruits is employed as a stomachic, hypoglycemic agent, and a antimicrobial substance. The plant is used to address issues such as coughs, oral diseases, and constipation, and it is used as an appetizer [24,25]. *M. communis* L. is rich in phenolic compounds like myricetine, gallic acid, vanillic acid, catechins, arbutin, and syringic acid [25]. Different authors have described the antioxidant activity of these extracts by introducing MCLE into food products. Amensour et al. [26] used *M. communis* L. water extract as a natural colorant, which was added to sausages to protect their color during long-term storage, allowing the iron cation to be in the reduced state (Fe^2+^) bound with myoglobin (in the presence of oxygen), giving oxymyoglobin, which gives the most characteristic color of fresh meat. This suggests that plant extracts have antioxidant properties that help prevent the oxidation of Fe^2+^ and, as a result, color degradation in meat products. Boroujeni et al. [27] used *M. communis* L. oil mixed with sunflower oil to prevent its oxidation. These findings indicated that the oil from *M. communis* L. served as an effective antioxidant when combined with other oils, helping to maintain the freshness and quality of the food product. 

This study aims to explore the potential of *M. communis* leaf extract (MCLE) to enhance the oxidative stability of oil-in-water emulsions. The first part of this study assesses the antioxidant and scavenging activities of MCLE under varying concentrations of ethanol (40%, 60%, and 80%). This will provide information on the most effective ethanol concentration for preserving the extract’s antioxidative properties. In order to help determine which extract will work best for use in future studies, this study also aims to identify and quantify the phenolic compounds that are present in MCLE at various ethanolic concentrations. The ultimate goal is to understand how MCLE, which is incorporated directly in spheres and an alginate film, influences the oxidation of oil-in-water emulsions and offers potential applications. Additionally, this study aims to investigate how the alginate films, which incorporate MCLE, interact with different food simulants. This research also aligns with the growing interest in natural and sustainable methods for food product preservation.

## 2. Materials and Methods

### 2.1. Chemicals and Standards

All the reagents were acquired from Sigma-Aldrich Química S.A. (Madrid, Spain). Anhydrous sodium carbonate (Na_2_CO_3_), Folin–Ciocalteu reagent, DPPH (2,2-diphenyl-1-picrylhydrazyl), methanol (CH_3_-OH), ethanol (C_2_H5OH), glacial acetic acid (CH_3_-COOH), aluminum trichloride hexahydrate (AlCl_3_·6H_2_O), sodium acetate trihydrate (CH_3_COONa·3H_2_O), hydrochloric acid 37% (HCl), iron chloride (III) hexahydrate (FeCl_3_ 6H_2_O, TPTZ (2,4,6-tripyridyl-s-triazine), ABTS (2,2-Azino-bis(3-ethylbenzothiazoline-6-sulfonic acid), potassium persulfate (K_2_S_2_O_8_), PBS (phosphate-buffered saline), AAPH (2,2-Azobis(2-methylpropionamidine) dihidrochloride 97%)), fluorescein sodium salt (C_20_H_10_O_5_Na_2_), Trolox 97% (6-hydroxy-2,5,7,8-tetramethylchroman-2-carbonsaeure 97%), Tween-20, methyl linoleate (C_19_H_34_O_2_), iron (II) chloride (FeCl_2_), and ammonium thiocyanate (NH4SCN).

The flowing standards epicatechin (C_15_H_14_O_6_), arbutin (C_12_H_16_O_7_), ferulic acid (C_10_H_10_O_4_), sinapic acid (C_11_H_12_O_5_), p-Coumaric acid (C_9_H_8_O_3_), cinnamic acid (C_9_H_8_O_2_), myricetine (C_15_H_10_O_8_), kaempferol (C_15_H_10_O_6_), vanillic acid (C_8_H_8_O_4_), and gallic acid (C_7_H_6_O_5_) were also acquired from Sigma-Aldrich Quimica S.A. (Madrid, Spain).

### 2.2. Extract Preparation

The plant *M. communis* L. was collected in September from the north of Morocco (35°46′48″ N and 5°54′36″ W). Afterward, the leaves were accurately separated from the rest of the plant and dried in the shade at a temperature of ~25 °C until they reached a constant weight (the final dry material of the samples was ~40.56% of the initial one). Then, the plant was crushed with a blender (Retsch SM100, Paris, France) and sieved (1 mm) (Gylson Company, Inc., Lewis Center, OH, USA) to obtain a homogeneous powder. The powder was kept in plastic boxes at −80 °C until use. 

Plant leaf extracts were obtained using ethanol/water at the following three different concentrations: 40, 60, and 80% (*v*/*v*). In all cases, 0.5 g of the plant powder was dissolved in 10 mL of the solvent at room temperature. The mixture was stirred in dark flask glasses using a multiposition magnetic stirrer (Ovan, MM90E, Barcelona, Spain) for 6 h at room temperature (~20 °C). The supernatant was collected after the extract had been centrifuged (OrtoAlresa Mod, Consul, Ajlvir, Madrid, Spain) for 10 min at 3000 rpm at ~24 °C. Plant extracts were kept in tinted glass vials at −80 °C until the corresponding analyses were performed.

### 2.3. Radical Scavenging Activity 

#### 2.3.1. Total Polyphenol Content (TPC)

The Folin–Ciocalteu technique was used to calculate the total polyphenol content (TPC) of all of the samples, according to Ourfelli et al. [28]. The previously obtained plant extract was placed in a microplate with 96 walls (20 µL). After this, 20% (m/V) sodium carbonate (80 µL) and the Folin reactive were added (80 µL). The plate was shaken in the microplate reader before being placed in the dark for one hour and adding ultrapure water (80 µL). The absorbance was determined at 725 nm by the micro-plate reader. TPC was determined by interpolating the absorbance of samples against a calibration curve, which was made using a standard solution of gallic acid at different concentrations (250 to 2000 µM) (R^2^ = 0.993). The results were presented as the mg gallic acid equivalent per mL of sample (mg GAE/mL of sample extract).

#### 2.3.2. Total Flavonoid Content

The total flavonoid content (TFC) of the extracts was measured by employing a chloride colorimetric assay, as reported by Odumusu et al. [29]. The amount of flavonoids was calculated by adding triclorure aluminum (50 µL) to the plant extract (150 µL, with an adequate dilution) and then reading the absorbance at 405 nm with a multimode micro-plate reader (FLUOstar^®^ Omega, Ortenberg, Germany). The TFC values were obtained according to a calibration prepared with quercetin at different concentrations (50 to 500 μM) (R^2^ = 0.995). The results were presented in mg quercetin equivalents per mL of sample (mg QE/mL of sample extract).

#### 2.3.3. Trolox Equivalent Antioxidant Capacity Assay

The Trolox equivalent antioxidant capacity (TEAC) assay was performed as described by Gallego et al. [30]. The absorbance was measured using a microplate reader (UV–vis spectrophotometer (FLUOstar OMEGA, Perkin-Elmer, París, France) whose temperature was fixed at 30 °C. The absorbance was measured at 734 nm, and the value after 7 min was chosen to determine the absorbance variation. The TEAC values were determined from a calibration curve made with Trolox at different concentrations (2 to 35 µM) (R^2^ = 0.996). The results were expressed in milli-mole of Trolox equivalents per mL of sample (mM TE/mL of sample extract).

#### 2.3.4. Ferric-Reducing Antioxidant Power (FRAP) Assay

The ferric-reducing antioxidant power (FRAP) assay was estimated following the method reported by Gallego et al. [30], with minor changes. The absorbance of the sample was measured with a microplate reader (FLUOstar OMEGA, Perkin-Elmer, París, France) at 593 nm and at a temperature of 30 °C. The values were determined from a calibration curve of Trolox at different concentrations (2 to 35 μM)(R^2^ = 0.997). The results were expressed as mmol of Trolox equivalents per mL of sample extract (mM TE/mL of sample extract). 

#### 2.3.5. 2,2-Difenil-1-Picrilhidrazil (DPPH) Assay

The DPPH assay was performed as described by Bevan et al. [31], with slight modifications. The reactive solution of DPPH was obtained by dissolving it in methanol. The absorbance was measured in a microplate reader (FLUOstar OMEGA, Perkin-Elmer, París, France) at 517 nm, and the result after 15 min was used to calculate the absorbance variation. The results were calculated with a calibration curve of Trolox at different concentrations (1 to 35 μM) (R^2^ = 0.992). The results were expressed as mmol of Trolox equivalents per mL of sample extract (mM TE/L of sample extract).

#### 2.3.6. Oxygen Radical Antioxidant Capacity (ORAC) Assay

The antioxidant activities of plant leaf extracts were determined using an ORAC assay, as reported by Gomez et al. [32], with minor changes. The absorbance was measured in a microplate reader (FLUOstar OMEGA, Perkin-Elmer, Paris, France) at 37 °C and every 2 min up to 120 min. The results were calculated with a calibration curve of Trolox at different concentrations (1 to 35 μM) (R^2^ = 0.995). The results were expressed as mmol of Trolox equivalents per mL of sample extract (mM TE/mL of sample extract).

### 2.4. Identification and Quantification of Phenolic Compounds by High-Performance Liquid Chromatography (HPLC-DAD)

The identification and quantification of the constituents of the samples were performed by HPLC analysis, according to Bevan et al. [31]. The assay was carried out using Waters alliance 2695 Series HPLC-DAD equipment (Waters, Wilmslow, UK). The equipment consists of an automatic sample injection system, two high-pressure pumps, a degasser, and a chromatographic oven. The chemical components were separated with a water Symmetry C18 column (150 mm × 3.9 mm, 5 m, Waters, UK). The HPLC-DAD conditions were set as follows: The mobile phase was composed of Phase A (ultrapure water acidified with 0.1% formic acid) and Phase B (acetonitrile acidified with 0.1% formic acid). The elution gradient corresponded to 95% A and 5% B, with a flow rate of 1 mL/min, injection volume of 25 µL, and room temperature (≈25 °C). Different commercial standards were subsequently used to identify the compounds detected by HPLC-DAD. The identification of the components was confirmed by matching their retention time (RT) and spectrum to those of the corresponding authentic standard compounds. The results were expressed as micrograms of phenolic compounds per liter of infusion. 

### 2.5. Antioxidant Activity Evaluation by HPLC-ABTS

The antioxidant activity of each compound of the plant extracts was assessed using the HPLC-ABTS radical post-column ABTS^+^ solution injection, as reported by Gomez et al. [32]. The method of HPLC described in Section 2.4 was followed, with the addition of a post-column [33] that comprised a pump to inject the ABTS radical. The ABTS^+^ injection has a flow rate of 0.4 mL/min. After the compounds immigrated into the column, it reacted with the ABTS radical, allowing the reaction of each compound with the cation radical and giving, at retention times where a compound with antiradical activity was present, a decrease in the absorbance due to the ABTS. The ABTS radical was guided in peek tubing that was 3 m long and had an internal diameter of 0.4 mm. The peaks were then detected using a DAD detector at wavelengths of 280, 320, and 734 nm. 

### 2.6. Spheres Preparation

Spheres were obtained as described by Bevan et al. [31] from the plant infusion by dissolving 0.5 g of the powdered plant in 10 mL of 60% (*v*/*v*) ethanol and calcium lactate. A semispherical mold was filled with 5 mL of the mixture, and it was frozen at −40 °C. For the spherfication, 5 g of alginate (Vinpai (Cimalgin, France)) was dissolved in deionized water to create an alginate bath. The bath was left overnight to remove the air bubbles. The frozen spheres were placed in the bath for 5 min. They were kept in vials that were filled with the same mixture to keep the spheres’ internal composition stable.

### 2.7. Film Preparation and Characterization: Difussivity Assay

#### 2.7.1. Preparation of Films

Alginate films were produced by dissolving 2.5 g of two different alginate types (1.25 g from each types (Alginate 6031 (Brenntag, Spain) and Vinpai (Cimalgin, France)) in 100 mL of distilled water. After eliminating the air bubble from the alginate, 30% (*v*/*v*) of the corresponding plant infusion was added. For 15 min, the mixture underwent stirring to form a homogeneous liquid. The liquid film (~13–15 g) was placed in a Petri plate (110 mm), and it was left to dry at room temperature (~48–72 h). The dried films were immersed for 5 min in 100 mL of a crosslinking solution (30% (*v*/*v*) of ethanol and 3% (*w*/*v*) of CaCl_2_), which was prepared according to the process described by Farias et al. [34]. After removing the extra solution, the films were dried for two hours at 25 °C. The films were stored on a petri plate until their use.

#### 2.7.2. Microstructure of the Films

The microstructure of the films was determined using scanning electron microscopy (SEM) (-J-7100-FE, Oxford Instruments, Oxford, UK). Small pieces of the films (8 × 8 mm) were fixed to the stubs directly, and images were observed by scanning the film surface, according to Odumosu et al. [29]. 

#### 2.7.3. Film Diffusivity

The diffusivity of the active compounds of the film was evaluated by placing small pieces of the film in different vials, which contained different food stimulants. In the current study, four food stimulants were used to assess this assay: ethanol 20% (*v*/*v*), ethanol 50% (*v*/*v*), MiliQ water, and acetic acid 3% (*w*/*v*). The films (~15 mg) were placed in different small vials, which contained 1 mL of each food simulant. Then, the vials were stored for 3 days at room temperature at ~20 °C. The simulant samples were collected at different times (every 10 min of the first hour, every hour during the first 4 h, and at 3 days) to evaluate the diffusion of the radical scavenging compounds from the films to the liquid. The antiradical activity of these samples was assessed according to the methods described in Section 2.3.

### 2.8. Oil-in-Water Emulsions: Antioxidant Activity

#### Preparation of Emulsions

The oil-in-water (*o*/*w*) emulsion was prepared as follows: Tween-20 (1% *w*/*w*), Milli-Q water (89% *w*/*w*), and methyl linoleate (MeLo, 10% *w*/*w*). Previously, Tween-20 and Milli-Q water were diluted by dispersing them using a magnetic stirrer. In an ice bath, MeLo oil was added to this mixture dropwise while being continuously sonicated for 5 min using an ultrasonic homogenizer (Hielscher, UP200S, Teltow, Germany) [31]. The product was distributed in different vials, and each vial was filled with ~13 g of emulsion. 

The following emulsions were elaborated: emulsions with spheres (one sphere in a vial), two emulsions with different amounts of plant infusion (1 mL and 1.5 mL of MCLE60), and one emulsion with films incorporating 30% of MCLE60 (~0.20 g of the film). Additionally, two controls were also employed: one was a negative control that only contained the emulsion with 1.5 mL of 60% ethanol, and the other was a positive control, which was obtained by the addition of 1.5 mL of a gallic acid solution (0.07% gallic acid solution, *w*/*w*). The vials were placed in an oven set to 25 °C and kept dark for 16 days. 

### 2.9. Study of the Antioxidant Activity in the Aqueous Phase of the Emulsions

To study the antioxidant activity in the aqueous phase of all the emulsions, a thermal shock was used to separate their two phases. After eliminating the films and spheres from the emulsion, the emulsion was frozen at −80 °C for 24 h, followed by immersion in a 90 °C bath. This process was repeated three times until complete separation of the two phases was achieved. The aqueous phase was then collected in vials and frozen for future use. The scavenging activity was determined following the procedure described in Section 2.3.

### 2.10. Oxidation Reactions 

#### 2.10.1. Primary Oxidation Measures (Peroxide Value, PV)

The primary oxidation of emulsions was determined by the peroxide value using ferric thiocyanate, as reported by Villasante et al. [35]. After combining emulsion drops (weighing exactly between 2 mg and 13 mg) with 1 mL of EtOH (98%), the mixture was completely homogenized using a vortex. This solution was then mixed with EtOH to achieve a final volume of 4 mL in a plastic cuvette. A total of 75 µL FeCl_2_ (in 37% HCl) and 75 µL ammonium thiocyanate were then added. The blank contained 4 mL of absolute EtOH and 1.8% (*v*/*v*) of each reactant. 

Analyses were performed using a UV spectrum absorbance spectrometer (FLUOstar OMEGA, Perkin-Elmer, París, France). Throughout the whole 14 days of the storage period, the absorbance was measured every 1–2 days at 500 nm. The results were expressed as milli-equivalents of hydroperoxides per kilogram of emulsion (meq hydroperoxides/kg emulsion). 

#### 2.10.2. Secondary Oxidation Reactions (Thiobarbituric Reactive Substances, TBARS)

The secondary oxidation reactions of lipids were evaluated according to the thiobarbituric reactive substances assay [36]. A total of 0.8 mL of TBARS reagent was added to 0.2 mL of each type of emulsion, and then samples were incubated in a water bath for 10 min. at a temperature of 96 °C. Then, vials were centrifuged (Orto Alresa Mod. Consul, Ajlvir, Madrid, Spain) at 2500 rpm for 5 min, and the supernatants of each tube were accurately removed and diluted to the appropriate reading range. The absorbance was measured at 531 nm using a UV–vis spectrophotometer (FLUOstar OMEGA, Perkin-Elmer, Paris, France). The results were expressed as mg of malonaldehyde per kg of emulsion (mg MDA/kg emulsion). 

### 2.11. pH Value

The pH value of emulsions was determined using a pHmeter (Criston Instruments, Barcelona, Spain). The measures were performed at room temperature.

### 2.12. Statistical Analysis

The results were statistically analyzed using the Minitab^®^ 17 software (Minitab, Inc., State College, PA, USA) by analysis of variance (ANOVA), and Tukey’s multiple comparison test was applied to determine the significant differences among samples (*p* < 0.05). Assays and measures were performed in triplicate, and the results are reported as means ± standard deviation.

## 3. Results and Discussion

### 3.1. Phenolic Profile and Scavenging Activity of M. communis L. Leaf Extracts

#### 3.1.1. Total Phenolic (TPC) and Flavonoid Content (TFC)

Table 1 presents the total phenolic and flavonoid contents of MCL extracts (MCLEs) at different ethanol concentrations (40, 60, and 80%). MCLE60 showed the highest TPC (~66.06 g GAE/L), which suggested that the extraction of phenolic compounds of MCLE with a solution of ethanol at ~60% improves the outcome. On the contrary, the lowest TPC corresponded to the sample obtained with 40% ethanol, with ~63.67 g GAE/L. However, the amount of TPC that these samples (MCLE40, MCLE60, and MCLE80) exhibited was similar. In all samples, TFC represented ~27–30% of total phenolic compounds (Table 1). In this case, the best extractive conditions for the flavonoids corresponded to the ethanol solutions at ≥60%, according to MCLE40 < MCLE60 = MCLE80 (*p* < 0.05). Variations in the phenolic content across different ethanol concentrations demonstrated that the selection of the concentration of the extraction solvent is an essential element, which influences their extraction efficiency from the plant extracts since the solvents possess varying affinities for these specific phytochemicals. In this context, the solvent’s polarity and its interactions with plant matrix components play a significant role [37].

#### 3.1.2. Radical Scavenging Activity of MCL Extracts

Table 2 shows the antiradical activity of MCL extracts. As observed, MCLE extracts obtained with ethanol concentrations of 60 and 80% exhibited a substantially higher scavenging activity in ABTS, DPPH, and ORAC assays (*p* < 0.05) than that observed in MCLE40. In the ABTS and DPPH assays, MCLE60 and MCLE80 showed values of 30.61–29.71 mmol TE/L and 28.75–26.62, respectively, while the 40% extract showed a lower activity (14.96 and 14.79 mmol TE/L in the ABTS and DPPH assays, respectively). In the ORAC assay, the 60 and 80% ethanol extracts displayed an antiradical activity of about 14.94 and 15.27 mM TE/L, respectively, contrasting with the 40% extract at 5.06 mmol TE/L. The FRAP assay further affirmed *M. communis* L. as a free radical inhibitor, with values of 24.85 mmol TE/L and 13.04 mmol TE/L for the 60% and 40% ethanol extracts, respectively, and higher activity (*p* < 0.05) in the 80% ethanol extract. Thus, according to these results, MCLE80 and MCLE60 exhibited the highest scavenging activity if compared with the MCL extract obtained at a lower concentration of ethanol (40%).

The study presented by Guzelmeric et al. [22], in which methanol was used as a solvent to extract the TPC of the whole plant (leaves, fruits, and flowers), also revealed an interesting capacity for *M. communis L.* to inhibit free radicals. In this study, in DPPH and ABTS assays, the authors showed an inhibiting capacity of the extract of 447.91 and 578.92 mg TE/g, respectively, and in the FRAP assay, they indicated a reducing activity of 257.27 mg TE/g. In a different study, which was presented by Tuberoso et al. [38], three different extracts of MCL (extracts obtained with ethanol, water, and ethyl acetate) were evaluated by measuring their antioxidant and antiradical activities. The ethanolic extract exhibited the best antioxidant potential, with an FRAP value of 84.7 mM TE/L and a DPPH value of 41.4 mM TE/L. The results presented collectively highlight the powerful antioxidant abilities of *M. communis* L., indicating its potential application as a natural antioxidant in various industries, and confirm the impact of the extraction methodologies on the bioactive characteristics of these extracts.

### 3.2. Phenolic Compounds in M. communis L. Extract

Table 3 presents the phenolic compounds identified in the MCLE by HPLC-DAD. As shown, eight compounds were detected in these plant extracts, the majority of which showed a higher amount in the extract obtained with ethanol at 60%.

In the present study, the principal compound identified in all of the samples corresponded to arbutin, followed by epicatechin. As observed, the content of arbutin increased (*p* < 0.05) in accordance with the percentage of ethanol in the extracting solution. Therefore, MCLE80 showed the highest amount of this compound, which corresponded to 155.16 mg/L of infusion. Epicatechin showed a higher presence in both ethanolic 60 and 80% extracts, registering at 73.89–75.64 mg/L, respectively, in comparison to MCLE40 (69.25 mg/L). Sinapic acid, gallic acid, kaempferol, and myrcetin compounds followed them in importance, which showed a higher amount in the 60% ethanolic extract (*p* < 0.05), with values of ~51.85 mg/L, ~23.76 mg/L, ~36.72 mg/L, and ~23.76 mg/L, respectively. In general, the lowest content of these compounds was also detected in the extract obtained with ethanol at 40% (Table 2). On the contrary, ferulic acid showed the highest amount in MCLE40, which corresponded to ~20.61 mg/L. Guzelmeric et al. [22] also reported the presence of myrcetin in the hydroalcoholic extract of *M. communis* L. (extract of leaves, flowers, and fruits) and demonstrated that the highest prevalence of this compound was found in the leaves (~25.01 mg/g in leaves), followed by the flowers (~11.14 mg/g) and the fruits (4.07 mg/g). Tuberoso et al. [38] reported results on the phenolic composition of MCLE using three solvents (ethanol, water, and ethyl acetate). The study revealed the presence of various compounds, including myrcetin, gallic acid, ellagic acid, and quercetin. The highest content was observed in the ethanol and ethyl acetate extracts, while the water extract had the lowest concentration. The concentrations of compounds were ~111.5 mg/L of gallic acid in the ethanolic extract, ~342.2 mg/L for myrcetin, ~36.2 mg/L for quercetin, and around 76 mg/L for ellagic acid. In the results reported by Snoussi et al. [39], the ethanolic extract (at 80% of ethanol) of the dry powder of *M. communis* L. leaves pointed toward gallic acid, myrcetin, and kaempferol as significant phenolic compounds in the sample, with concentrations of ~0.98, ~0.38, and ~0.12 mg/g, respectively. Aidi Wannes et al. [40], who worked with methanolic extracts, also revealed the occurrence of the same compounds in the MCLE, including myrcetin, gallic, vanillic, and ferulic acids (with amounts of 0.10, 1.15, 0.04 mg/g, and 0.05 mg/g, respectively). As observed, these results highlighted the importance of these plants as sources of diverse flavonoids, phenolic acids, and tannins. These substances are well known for their wide range of applications, especially as food preservers [41]. The quantity of these elements in the plant extract depends on various factors, of which the selection of the solvent and its concentration is one of the most important [42]. 

### 3.3. Antioxidant Activity Evaluation by HPLC-ABTS

According to the results observed previously, the antioxidant activity evaluation by HPLC-ABTS was assessed only in MCLE60. MCLE60 was selected due to the higher amount of compounds extracted at this percentage of ethanol. Table 4 presents the quantification of the standards (commaric acid, epicatechin, gallic acid, and myercetin), which were identified in MCLE60. The determination of the antioxidant activity was based on a decrease in the absorbance after the post-column reaction of the separated antioxidants with the radical cation ABTS^+^. This comprehensive approach allows for an accurate assessment of the antioxidant potential of known compounds present in the herbal material, including determining their individual concentrations and contributions to the general antioxidant properties.

As described previously (Table 3), the prevalence of these phenolic compounds corresponded to epicatechin > gallic acid > myrcetin > commaric acid, which were the dominant free radical scavengers of this extract. In general, these results again demonstrated the free radical scavenging activity of MCLE60 extracts. To calculate the third column, i.e., the amount of antiradical activity corresponding to the area of the negative peak, a calibration line was made using three concentrations of each of the standards to analyze the negative area compared to the concentration. In this way, it was possible to compare the contribution of each of the compounds to the total antiradical activity of the ethanolic extract.

### 3.4. Alginate-Based Films and Spheres Reinforced with MCLE Extracts

#### 3.4.1. Film Characterization

SEM was employed to analyze the microstructure. The surface scanning revealed microstructural differences in the film, including the control film without the presence of MCLE, as well as modifications happening before and after the introduction of crosslinking solutions. 

**Figure 1:**
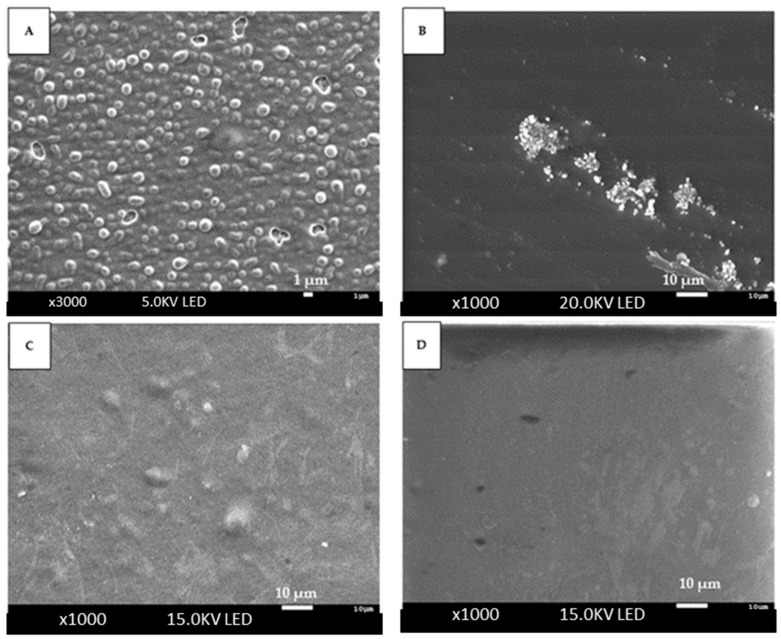
Film surface images obtained by scanning electron microscopy (SEM). (**A**) Alginate film. (**B**) Alginate film with crosslink solution (30% (*v*/*v*) of ethanol and 3% (*w*/*v*) CaCl_2_). (**C**) Alginate film with 30% plant extract. (**D**) Alginate film with 30% plant extract and crosslinking solution (30% (*v*/*v*) of ethanol and 3% (*w*/*v*) CaCl_2_).

Figure 1 presents the images of the film surfaces. As observed in Figure 1A, the film elaborated only with alginate showed a surface that had different pores, and it was non-homogeneous. After immersing the film in the crosslinking solution, the surface became more compact, smoother, and more homogenous (Figure 1B). As observed, the microstructure of the films changed when the plant extract was added to the solution. The incorporation of the *M. communis* L. extract into the film also resulted in a less homogeneous surface, with visible pores (Figure 1 C), although after putting the film on the crosslinking solution (Figure 1D), it showed a smoother and more homogeneous surface. 

Nevertheless, the films elaborated with the plant extract, alginate, and crosslinking solution showed some cracks and pores, through which the bioactive components of MCLE could be released from the film to the environment. 

#### 3.4.2. Diffusivity Assay

**Figure 2:**
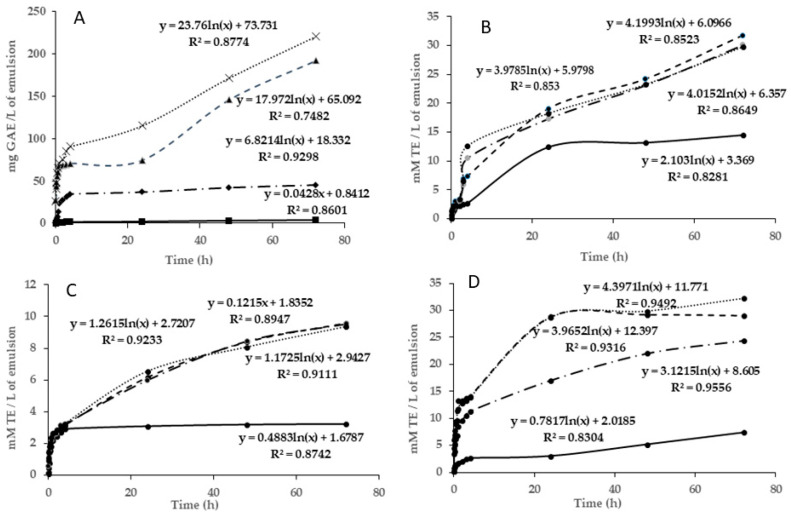
Evaluation of diffusivity of bioactive compounds from the film with *Myrtus communis* L. extract to different stimulants during 72 h. Simulants: (—•) ethanol, 20%; (—) ethanol, 50%; (---) H_2_O; (…) acetic acid. (**A**) Total phenolic compounds. (**B**) DPPH assay. (**C**) FRAP assay. (**D**) ABTS assay. TE: Trolox equivalent; GAE: gallic acid equivalent.

Figure 2A shows the release of TPC from the alginate films with MCLE60 extract in different food simulants (ethanol 20% (*v*/*v*), ethanol 50% (*v*/*v*), MiliQ water, and acetic acid 3% (*w*/*v*)). The scavenging activity of these samples was also presented in this figure. As observed, the total phenolic compounds revealed distinct patterns according to the type of simulant. Within the initial 5 min, the film that was in contact with water and acetic acid exhibited a notably higher release of the total phenolic compounds, surpassing ~25 mg GAE/g, while those in contact with ethanol at 20 and 50% showed lower concentrations of TPC (of ~2 mg GAE/g). A higher polarity of water and acetic acid favored the diffusivity of TPC in both mediums. For this reason, the release of TPC increased rapidly over time in acetic acid and water simulants, whereas in both ethanol simulants, the release of phenolic compounds remained more stable (see Figure 2). After 72 h, the total phenolic compounds measured in water and acetic acid corresponded to 191.88 and 220.77 mg GAE/g, respectively. Additionally, Figure 2A shows that with time, the diffusivity tendency in ethanolic simulants was linear, whereas in the water and acetic acid simulants, it exhibited a logarithmic trend, which indicated larger diffusiveness per time after the first hours of storage. Also, the reaction between the films and the food stimulant affected the surface of the film, which, in certain cases, became more permeable. This increase in permeability probably facilitated the passage of phenolic compounds from the film to the food stimulants.

All simulants were also evaluated for their scavenging activity by DPPH, FRAP, and ABTS assays. The results are shown in Figure 2B,D. Concerning the DPPH assay (Figure 2B), films in acetic acid and water simulants experienced the highest slope after the first hours of storage (reaching ~20 mmol TE/g after 1 h of storage), which could be attributed to the better antiradical efficiency of these matrices with a DPPH∙ radical. The ethanolic simulant at 20% showed similar behavior, whereas the ethanolic simulant at 50% maintained stable antiradical activity during the storage time (4.5 mmol TE/g). The ABTS assay confirmed these results, highlighting water and acetic acid simulants as those that presented the best antiradical capacity (*p* < 0.05) (Figure 2D). The higher slope obtained in these samples indicated that in these two mediums there was the highest antiradical capacity to inhibit the ABTS^+^ radicals. According to the logarithmic trends, the FRAP method (Figure 2C) has the best results for the diffusivity of the biochemical compounds to the simulants. This is indicative of the fact that the compounds diffused into the medium have more antioxidant (Fe^+2^ protective) capacity than antiradical activity. The results observed from this method demonstrated again the worst antiradical capacity of the ethanolic simulant at 50%. These results could be explained by the higher phenolic content of the water and acetic simulants (Figure 2A), followed by ethanolic simulants at 20 and 50%. These findings indicate that these two environments enhanced the film permeability, facilitating the transfer of the antioxidant compounds and, consequently, improving the radical scavenging capacity of the matrix. The best scavenging activity detected in all simulants after the first hours of storage could be explained by the highest diffusion of TPC from the films at the beginning.

The study presented by Dordevic et al. [43] analyzed the incorporation of blueberry, parsley, and grape extracts into biodegradable films to evaluate their physical and chemical properties. An antioxidant activity analysis revealed that polyphenolic compounds moved when these films were exposed to a food simulant (ethanol, 10%). The addition of extracts from parsley, red grapes, and blueberries resulted in greater antioxidant levels (in the films with 20% grape extract, the DPPH results were 53.15%, the 20% blueberry extract films showed a DPPH value of 70.07%, and the parsley film extract showed 58.75%). This suggests that these extracts have a substantial capacity for antioxidants, probably because of their high polyphenol content.

The release of phenolic compounds from alginate films offers practical applications beyond research in food packaging with these films, releasing natural preservatives for longer shelf life. This controlled release enhances efficacy, reduces dosage, and improves stability [44,45].

### 3.5. Application of Films and Spheres in Oil-in-Water Emulsions

#### 3.5.1. pH Value Results

The pH value of the different emulsions was checked over 14 days of their storage, which remained stable in all products. The pH value of all samples was similar (*p* > 0.05), and it corresponded to 2.66 ± 0.08 for the control emulsion, 2.66 ± 0.08 for the emulsion with gallic acid, 3.43 ± 0.04 for the emulsion with spheres, 2.73 ± 0.07 for the emulsion with the film, and 2.97 ± 0.07 and 3.01 ± 0.04 for the emulsions with 1 mL and 1.5 mL of MCLE60, respectively. These low values of pH could influence the loss of antioxidant efficacy over time due to the acceleration of lipid oxidation reactions. 

#### 3.5.2. Primary Oxidation Products (Peroxide Value)

In this study, the primary oxidative stability of the emulsions was examined by measuring their peroxide value (PV) during a storage period of 14 days. Changes in the oxidative stability of emulsions were evaluated in different samples, which contained different quantities of MCLE60 (1 and 1.5 mL) and alginate spheres and films.

**Figure 3:**
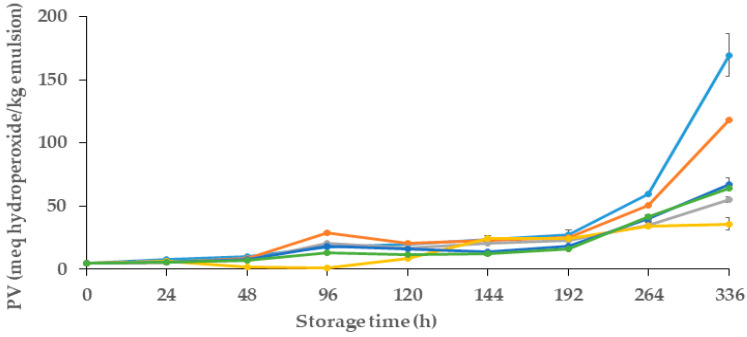
Evaluation of peroxide value (PV) of oil-in-water (O/W) emulsions during 14 days of storage. Control (●): emulsion; Control+ (●): emulsion with Gallic acid; Film (●): emulsion with alginate film made from alginate and 60% ethanolic extract of *M. communis* L.; Infusion 1.0 mL (●): emulsion incorporated with 1 mL of the 60% ethanolic extract of *M. communis* L.; Infusion 1.5 mL (●): emulsion incorporated with 1.5 mL of the 60% ethanolic extract of *M. communis* L.; (●): emulsion with spheres made from 60% ethanolic extract of *M. communis* L. PV: peroxide value; meq hydroperoxides/kg emulsion: milliequivalents hydroperoxides per kilogram of emulsion.

Figure 3 presents the evolution of the peroxide value of samples over their storage period. For all samples, in the initial 48 h, PV was similar (*p* > 0.05) and remained consistently low, with values less than ~10 meq hydroperoxides/kg of emulsion. After that, between 48 and 192 h (8 days), a gradual increase in the PV was observed in all emulsions, which presented significant differences in some storage points (*p* < 0.05). As observed, both control samples (control− and control+) showed the highest amount of PV after 8 days of storage (with 27.16 and 25.32 meq hydroperoxides/kg emulsion, respectively). After 14 days of storage (336 h), and as expected, the control− showed a lower oxidative stability than the control+, which could be attributed to the presence of gallic acid in the control+, which reduced the adverse primary oxidative reactions in that sample.

As shown in Figure 3, emulsions that contained MCLE showed better oxidative stability in comparison to both controls, even whit the positive control used (gallic acid). Regarding the primary oxidation reactions, emulsions with spheres showed the best oxidative stability, with a PV of 35.77 ± 0.5 meq hydroperoxides /kg of emulsion. Followed by the emulsion with alginate films that showed a PV of 55.08 ± 0,27 meq hydroperoxides/kg emulsion, followed by the emulsions with the incorporation of 60% ethanolic extracts, with values of 64.09 ± 0.5 and 67.34 ± 0.7 meq hydroperoxides/kg emulsion) for 1.5 mL and 1 mL infusions, respectively. No statistical differences (*p* > 0.05) were observed between the oxidative stability of these two samples (infusions with 1 and 1.5 mL of extracts). These results indicate the potential of MCLE60 to enhance the oxidative stability of oil-in-water emulsions, signifying promising avenues to improve their shelf life and quality. 

In line with this, Ouerfelli et al. [28] also reported the antioxidant effects of *Azadirachta indica* leaf extracts and improvements to the oxidative stability of emulsions. Their results also demonstrated that emulsions that had two different concentrations of the plant leaf extracts (0.25 and 0.5% of leaf extract, which were obtained with 50% aqueous EtOH) showed lower peroxide values (51.02 and 151.63 meq hydroperoxides/kg, respectively) after 30 days of storage in comparison to the control (emulsion without extract, which showed ~300 meq hydroperoxides/kg emulsion). In the study presented by Bevan et al. [31] about the effect of the incorporation of spheres with *Hibiscus sabdariffa* L. extracts in oil-in-water emulsion, the results showed important oxidative stability in the vials with 10 and 20 spheres. The peroxide values were around 50 meq hydroperoxides/kg emulsion after 240 h of storage; however, the control emulsion was 236 meq hydroperoxides/kg emulsion. These results reinforce the fact that the use of spheres as a delivery system enables the slow diffusion of the substances in the O/W emulsion to protect it from oxidation over the days of storage. 

#### 3.5.3. Secondary Oxidation Products

**Figure 4:**
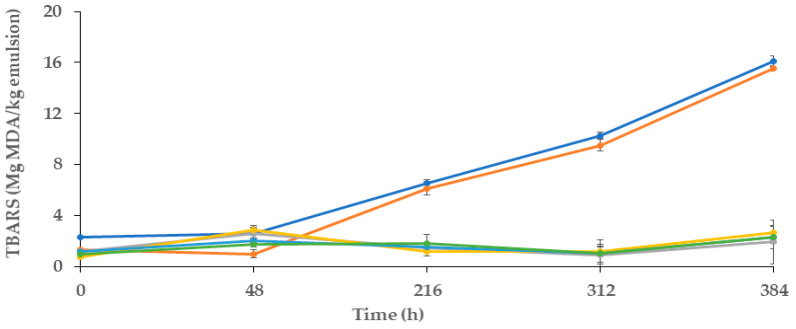
Evaluation of TBARS of oil-in-water (O/W) emulsions during 16 days of storage Control (●): emulsion; Control+ (●): emulsion with Gallic acid; Film (●): emulsion with alginate film made from alginate and 60% ethanolic extract of *M. communis* L.; Infusion 1.0 mL (●): emulsion incorporated with 1mL of the 60% ethanolic extract of *M. communis* L.; Infusion 1.5 mL (●): emulsion incorporated with 1.5 mL of the 60% ethanolic extract of *M. communis* L.; (●): emulsion with spheres made from 60% ethanolic extract of *M. communis* L. MDA: Malonaldehyde.

Figure 4 presents the results of the secondary reactions of oxidation of these emulsions, which were assessed during the 16 days of storage.

As expected, before the first 48 h of storage, the values of TBARS for all of the samples were not significantly different, and all of them < 3 mg MDA/kg emulsion, (*p* > 0.05). The antioxidants from MCLE60 probably inhibited the breakdown of hydroperoxides into TBARS. However, after the first 48 h, the TBARS for the two control samples increased (*p* < 0.05). At this point, the control showed values of 2.32 mg MDA/kg emulsion, which at the end of the storage period (16 days) corresponded to 16.09 mg MDA/kg emulsion. During this time, the content of MDA in the control+ sample (emulsion with gallic acid) was similar to that observed in the negative control, which presented a lower value in the first 48 h (*p* < 0.05) (of ~1.31 mg MDA/kg emulsion), but by the end of the storage time (~15.54 mg MDA/kg emulsion), this value did not differ from the negative control (*p* > 0.05). These findings suggested that secondary reactions of oxidation in both samples occurred in a similar way. As observed in Figure 4, emulsions with MCLE60 (films, spheres, and samples with 1 and 1.5 mL of MCLE60 infusion) presented the lowest values of TBARS. In all cases, these values were lower than 2.6 mg MDA/kg of emulsion, and they did not differ significantly (*p* < 0.05) among them. Similar to the results found in primary reactions of oxidation evaluation, MCLE60 showed efficacy in preventing secondary reactions of oxidation in O/W emulsions, but in this case, no differences were observed between them, either directly including the plant extract in the sample or including it previously in spheres or the alginate film. 

In the study presented by Gallego et al. [21], the researchers explored the effects of *Caesalpinia decapetala* (CD) leaf extract on the oxidative stability of oil-in-water emulsions. Following a 4-week storage period, emulsions containing CD extract exhibited enhanced oxidative stability, as evidenced by a 50% reduction in the (TBARS) value, which is indicative of diminished lipid oxidation. 

Another study by Bevan et al. [31] investigated the encapsulation of Hibiscus sabdariffa (HS) plant leaves and their application to improve the oxidative stability of oil-in-water emulsions. The encapsulated HS extract, when added to the emulsion, effectively reduced the formation of malonaldehyde, a marker for oxidative damage. After 300 h of storage, the emulsions with encapsulated HS spheres demonstrated a notable 20% reduction in oxidation. In summary, both studies suggest that plant extracts, in various forms, have the potential to protect emulsions from oxidative deterioration.

Encapsulating MCLE in diverse matrices like liposomes or chitosan nanoparticles could improve its stability and targeted delivery [46]. In this sense, exploring encapsulation for improved stability and delivery and testing efficacy in complex food matrices like meat and dairy highlight the future research directions that would significantly enrich the current study and advance the potential of MCLE in the food industry. Examining their efficacy in foodstuffs is an appropriate and complementary methodology for the study of antiradical activity [47].

For all of the above reasons, the research carried out in this paper (exploration of diverse and innovative delivery systems, such as the encapsulation of plant extracts in films and spheres) holds significant promise in simplifying the incorporation of natural substances into various products. This approach not only enhances the ease of use but also expands the potential applications of plant extracts in different food products.

#### 3.5.4. Radical Scavenging Activity of the Aqueous Phase of the Emulsions

**Figure 5:**
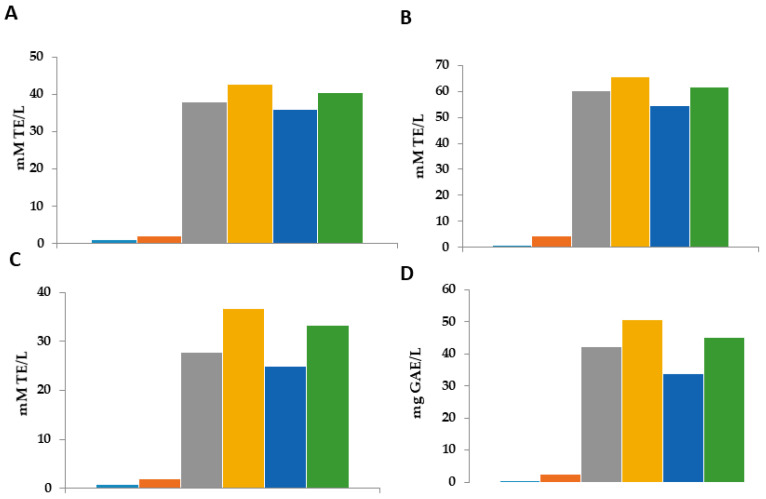
Evaluation of radical scavenging activity the aqueous phase of oil-in-water (O/W) emulsions. Control (●): emulsion only; Control+ (●): emulsion with Gallic acid; Film (●): emulsion with alginate film made from alginate and 60% ethanolic extract of *M. communis* L.; Infusion 1.0 mL (●): emulsion incorporated with 1 mL of the 60% ethanolic extract of *M. communis* L.; Infusion 1.5 mL (●): emulsion incorporated with 1.5 mL of the 60% ethanolic extract of *M. communis* L.; (●): emulsion with sphere made from 60% ethanolic extract of *M. communis* L. (**A**): DPPH, (**B**): FRAP, (**C**): ABTS, (**D**): Total Phenolic Compounds; TE: Trolox equivalent; GAE: gallic acid equivalent.

Figure 5 presents the radical scavenging activity and the antioxidant activity in the aqueous phase of the emulsion after breaking the emulsion in two phases using thyermique choc.

In order to evaluate the scavenging and antioxidant activity released in emulsions by various MCLE products, a study was performed on the aqueous phase of broken emulsions after their storage time. Four experiments (Figure 5) revealed that the emulsions containing any MCLE product had the highest scavenging activity, which was expected and in line with the previously noted oxidative stability. The results of the antiradical activity founded in the aqueous phase of the emulsion that had the MCLE60 (with the different forms) showed important results in the four different methods, which confirm the presence of antioxidant and phenolic compounds that were released in the emulsion from the MCLE (spheres, films, and extract). The results were significantly different (*p* = 0.05). The results in the different techniques were the same: the aqueous phase of the emulsion that was in contact with the spheres showed a higher scavenging activity, followed by the film and 1.5 mL of the MCLE60. Specifically, the aqueous phase of emulsions containing spheres demonstrated the highest antioxidant activity at around ~50 mg GE/L. Furthermore, spheres presented the strongest scavenging activity in all three assays; the slow release of the substances from the spheres to the emulsion could be the reason for those higher results that were founded in the films. In addition, the amount of MCLE60 encapsulated in the spheres could be the reason behind this important foundation. These results confirm that spheres are one of the most efficient ways to minimize emulsion oxidation, and they are in line with the previous study concerning oxidative stability.

The two controls showed the lowest scavenging activity; however, the results of the aqueous phase of the emulsion with any MCLE product showed better results: spheres > films = 1.5 mL MCLE > 1 mL MCLE. The results presented in this study are in line with the results presented in the previous study (Section 3.5.2 and Section 3.5.3). The presence of an important amount of antioxidant released by the MCLE in the emulsion is responsible for the oxidative stability of the emulsion.

## 4. Conclusions

This study revealed that *M. communis* L. leaf extract has noteworthy antioxidant activity. The extraction of TPC improved with a solution of ethanol at 60%, with ~66.06 g GAE/L. The antioxidant activity and scavenging activity of the ethanolic extract 60% were better than those of MCLE40 and did not show a significant difference with MLCE80. In all extracts, the flavonoids represented ~27–30% of the total phenolic compounds. Eight compounds were detected in these extracts, of which highlighted arbutin, epicatechin, gallic acid, and sinapic acid. Thus, according to these results, MCLE80 and MCLE60 exhibited the highest scavenging activity (FRAP, DPPH, ABTS, and ORAC), if compared with the MCL extract obtained at a lower concentration of ethanol (40%). Films elaborated with MCLE60, alginate, and a crosslinking solution resulted in a homogeneous surface with some cracks and pores. The diffusivity of TPC through films from different food simulants (ethanol at 20% and 50%; water and acetic acid at 3%) revealed a higher release in the water and acetic acid environments, probably due to their higher polarity. In consequence, these two simulants showed the best radical scavenging activity in comparison to the ethanol. Oil-in-water emulsions that contained MCLE60 (incorporated in films and spheres) exhibited the best oxidation stability, for more than a week, stored at a temperature of 30 °C. The inclusion of different delivery systems (spheres and films) with plant extracts in the emulsion also enhanced the primary and secondary reactions of oxidation in comparison to emulsions in which this extract was mixed directly. Thus, this study stated the potential uses of MCLE as a natural ingredient for the preservation of *o*/*w* emulsions and the production of active alginate films.

## Figures and Tables

**Table 1:** Total phenolic compounds (TPCs) and total flavonoid content (TFC) of *Myrtus communis* L. leaf extracts (MCLEs) obtained with different concentrations of ethanol (40, 60, and 80%).

Compounds ¹	*M. communis* L. Leaf Extracts ^2^		
	MCLE40	MCLE60	MCLE80
TPC (g GAE/L)	63.67 ± 0.02 ^c^	66.06 ± 0.11 ^a^	64.18 ± 0.05 ^b^
TFC (g QE/L)	17.36 ± 0.05 ^b^	18.91 ± 0.09 ^a^	18.74 ± 0.02 ^a^

^1^ TPC: total phenolic content; TFC: total flavonoid content; GAE: gallic acid equivalent; QE: quercetin equivalent. ^2^ The results are presented with the mean ± standard deviation. ^a–c^ In the same raw, different superscripts indicate significant differences (*p* = 0.05). MCLE: *M. communis* L. leaf extract obtained with three different ethanol concentrations (40, 60, and 80%).

**Table 2:** Radical scavenging activity of *M. Communis* L. leaves (MCL) extracts in different ethanolic solvents (40, 60, and 80%) determined by the ABTS, FRAP, DPPH, and ORAC assays.

Antiradical Methods (mmol TE/L) ^1^	*M. communis* L. Leaf Extracts ^2^		
	MCLE40	MCLE60	MCLE80
FRAP	13.04 ± 0.03 ^c^	24.85 ± 0.07 ^b^	32.21 ± 0.05 ^a^
DPPH	14.79 ± 0.05 ^b^	28.75 ± 0.06 ^a^	26.62 ± 0.07 ^a^
ABTS	14.96 ± 0.02 ^b^	30.61 ± 0.04 ^a^	29.71 ± 0.06 ^a^
ORAC	5.06 ± 0.07 ^b^	14.94 ± 0.08 ^a^	15.27 ± 0.05 ^a^

^1^ FRAP: ferric ion reducing antioxidant power; DPPH: 2,2-diphenyl-1-picrylhydrazyl assay; ABTS; 2,2-azino-bis-3-ethylbenzothiazoline-6-sulphonic acid assay; ORAC: oxygen radical absorbance capacity. ^2^ The results are presented with the mean ± standard deviation. ^a–c^ The letters in the same raw indicate significant differences (*p* < 0.05). MCLE: *M. communis* L. leaf extract obtained with three different ethanol concentrations (40, 60, and 80%).

**Table 3:** Amounts of different phenolic compounds detected in leaf extract of *M. communis* L.

Compound (mg/L of Infusion)	*M. communis* L. Leaf Extracts ^1^		
	MCLE40	MCLE60	MCLE80
Arbutin	68.14 ± 0.15 ^c^	122.08 ± 0.17 ^b^	155.16 ± 0.09 ^a^
Commaric acid	20.31 ± 0.01 ^a^	18.56 ± 0.01 ^b^	20.4 ± 0.26 ^a^
Epicatechin	69.25 ± 0.03 ^b^	73.89 ± 0.03 ^ab^	75,64 ± 0.14 ^a^
Ferulic acid	20.61 ± 0.01 ^a^	16.08 ± 0.01 ^b^	13.85 ± 0.04 ^c^
Gallic acid	33.81 ± 0.01 ^c^	36.72 ± 0.03 ^a^	34.98 ± 0.05 ^b^
Kaempferol	20.23 ± 0.01 ^c^	23.76 ± 0.02 ^a^	22.56 ± 0.02 ^b^
Myrcetin	22.19 ± 0.06 ^b^	23.84 ± 0.04 ^a^	21.29 ± 0.04 ^c^
Sinapic acid	44.19 ± 0.04 ^b^	51.85 ± 0.06 ^a^	32.12 ± 0.04 ^c^

^1^ The results present the mean ± standard deviation; ^a–c^ the letters in the same raw indicate significant differences *(p* < 0.05). MCLE: *M. communis* L. leaf extract obtained with three different ethanol concentrations (40, 60, and 80%).

**Table 4:** The antioxidant activity evaluated by HPLC-ABTS of some phenolic compounds of *M. communis* L. extract obtained with an ethanolic solution at 60%.

Compounds ¹	Retention Time (min.)	Positive Peaks	Negative Peaks
		Content (mg/L)	Antioxidant Activity (mg GAE/L)
Commaric acid	13.3	19.43 ± 0.08 ^c^	0.98 ± 0.01 ^c^
Epicatechin	11.02	71.32 ± 0.03 ^d^	5.99 ± 0.04 ^c^
Gallic acid	4.4	36.11 ± 0.13 ^a^	1.31 ± 0.03 ^a^
Myrcetin	15.2	22.97 ± 0.09 ^b^	1.21 ± 0.03 ^b^

¹ The results present the mean ± standard deviation. ^a–d^ Different letters in the same column indicate significant difference (*p* < 0.05) between the samples. GAE: gallic acid equivalent.

## Data Availability

Data are contained within the article.
